# Markov Chain-Like Quantum Biological Modeling of Mutations, Aging, and Evolution

**DOI:** 10.3390/life5031518

**Published:** 2015-08-24

**Authors:** Ivan B. Djordjevic

**Affiliations:** Department of Electrical and Computer Engineering, College of Engineering, University of Arizona, 1230 E. Speedway Boulevard, Tucson, AZ 85721, USA; E-Mail: ivan@email.arizona.edu; Tel.: +1-520-626-5119; Fax: +1-520-626-3144

**Keywords:** quantum biology, bioinformatics, DNA quantum information, biological channels, mutations, aging, evolution, channel capacity

## Abstract

Recent evidence suggests that quantum mechanics is relevant in photosynthesis, magnetoreception, enzymatic catalytic reactions, olfactory reception, photoreception, genetics, electron-transfer in proteins, and evolution; to mention few. In our recent paper published in *Life*, we have derived the operator-sum representation of a biological channel based on codon basekets, and determined the quantum channel model suitable for study of the quantum biological channel capacity. However, this model is essentially memoryless and it is not able to properly model the propagation of mutation errors in time, the process of aging, and evolution of genetic information through generations. To solve for these problems, we propose novel quantum mechanical models to accurately describe the process of creation spontaneous, induced, and adaptive mutations and their propagation in time. Different biological channel models with memory, proposed in this paper, include: (i) Markovian classical model, (ii) Markovian-like quantum model, and (iii) hybrid quantum-classical model. We then apply these models in a study of aging and evolution of quantum biological channel capacity through generations. We also discuss key differences of these models with respect to a multilevel symmetric channel-based Markovian model and a Kimura model-based Markovian process. These models are quite general and applicable to many open problems in biology, not only biological channel capacity, which is the main focus of the paper. We will show that the famous quantum Master equation approach, commonly used to describe different biological processes, is just the first-order approximation of the proposed quantum Markov chain-like model, when the observation interval tends to zero. One of the important implications of this model is that the aging phenotype becomes determined by different underlying transition probabilities in both programmed and random (damage) Markov chain-like models of aging, which are mutually coupled.

## 1. Introduction

In 1944 Erwin Schrödinger published a book entitled *What is Life?* [1,2]. In Chapter 1 of his book, Schrödinger introduces the *order-from-disorder principle* and explains that most physical laws on a large scale originate from the chaos on a small scale. In Chapter 4, he connects the mutations to the quantum jumps (leaps). In the same chapter, he explains that stability of molecules, being composed of atoms, should be attributed to the quantum mechanics. In Chapter 5 of his book, Schrödinger introduces the concept an “aperiodic crystal”, now known as DNA, which contains the genetic information in its configuration of covalent chemical bonds, and allows us to encode an almost infinite number of possibilities with a small number of basic units, now known as nucleotides. In addition to explaining DNA structure, Watson and Crick suggested that point mutations might be caused by tautomeric forms of nucleic acids [3]. This idea was later studied in a series of papers [4,5,6], and it is a subject of interest even these days [7,8]. In several recent publications [9,10], it has become evident that both the Darwinian type of evolution (random mutations followed by selection process) and the Lamarckian type of evolution (selected mutations beneficial to the organism) are important. Moreover, Cooper [11] has shown that evolutionary pressure has selected quantum probability laws over classical kinetics laws. In the same paper, Cooper also discusses the relevance of time-dependent substitutions and time-dependent deletions, indicating that the biological channel is a channel with memory.

The use of both classical and quantum information theory, as well as error-correction, to describe the genome preservation and biological evolution is getting momentum, which can be judged by a number of recent papers related to these problems [8,9,10,11,12,13,14,15]. There have been many attempts to explain the transfer of genetic information from DNA to protein by using the concepts of quantum mechanics [14,15]. However, the determination of a quantum biological channel capacity was still an open problem until recently [8].

In our recent paper [8], we have derived the operator-sum representation of biological channel based on codon basekets, and used this representation to determine the quantum channel model suitable for study of the quantum biological channel capacity. Unfortunately, this model is essentially memoryless and as such it can be used to determine the quantum genetic channel capacity for a given codon nucleobase error probability. However, this model is not able to properly describe the propagation of mutation errors in time, the process of aging, and evolution of genetic information through generations.

To solve for these problems, in this paper, we propose novel quantum mechanical models to accurately describe the process of creation spontaneous, induced, and adaptive mutations and their propagation in time. These models are based on an operator-sum representation [8] of the quantum biological channel. We propose the following quantum biological channels with memory: (i) Markovian classical model, (ii) Markovian-like quantum model, and (iii) hybrid quantum-classical model. Then we study how the quantum channel capacity changes in different stages of life, in other words, by aging. We then apply these models to study how the quantum biological channel capacity changes through generations. Further, we calculate quantum biological channel capacities for a multilevel symmetric channel (MSC) [16] based Markovian model and a Kimura model [17] based Markovian process, which serve as reference cases. As indicated in the abstract, these models are quite general and applicable to many open problems in biology, not only biological channel capacity, the main focus of the paper. As an illustration, we will demonstrate that the famous quantum Master equation approach, commonly used to describe different biological process, is just the first-order approximation of the proposed quantum Markov chain-like model, when the observation interval tends to zero.

The proposed models are also compatible with both damage/error and programmed theories of aging, reviewed by Jin [18]. The proposed Markovian-like quantum biological channel model can also be used to unify damage and programmed senescence theories of aging as follows. The aging process can be described by two Markov chain-like processes running in parallel. The first one is a programmed aging model, based on a life clock, and it describes the transition from one stage of life to another. When one particular transition probability in the model is close to 1 that means that the corresponding transition is deterministic. The transition probabilities in this model are more certain, but they are not completely deterministic; instead, they get perturbed by the environment. Transition from one stage in life to the next stage in life is determined by particular gene activation and deactivation. The second Markov chain-like model is the random aging Markov chain-like model that describes the change in biological channel capacity that results from deferent “genetic noise” errors. (For detailed description of various sources of genetic noise an interested reader is referred to reference [8].) It runs between two transitions in the programmed Markov chain-like model of aging. Therefore, the first one serves as a sort of control mechanism.

This has a number of testable implications for the biology of aging and for evolution. One interesting application to use this unified (programmed-damage) Markov chain-like model is to predict predominant types of error in the aging process of particular tissue or organ. The biological models described in this paper can also be used to determine, for instance, the death rate at a certain age. The programmed Markov chain-like model, as we indicated above, serves as a control mechanism that is not perfectly stable, but can be perturbed by environment, stress, lifestyle, habits, and random mutations, to mention few. The damage model operating between two stages in the programmed model can either slow down certain biological processes or speed them up. The interplay between these two models is not trivial to determine and requires further study, since the aging phenotype becomes determined by different underlying transition probabilities in both programmed and random Markov chain-like models. Given that the proposed Markovian-like quantum model is quantum stochastic and that the superposition principle is not used in its derivation, this model is consistent with recent finding that quantum mechanics is essentially nonlinear [19,20].

The paper is organized as follows. In Section 2, we describe the proposed quantum channel models suitable for study of mutations’ propagation in time and aging. We calculate how channel capacity changes in different stages of the life of the organism. In Section 3, we apply these quantum biological models and study the evolution of quantum channel capacity through generations. Corresponding MSC-based and Kimura model-based Markovian processes are also described and the corresponding evolution of capacity is evaluated as well. Section 4 concludes the paper.

## 2. Markovian Chain-Like Quantum Mechanical Modeling of Mutations and Aging

In this section we extend the memoryless quantum biological model, described in our previous paper [8], to a corresponding model with memory. Before we describe the proposed model, we briefly review the density operator concept and operator-sum representation. The density operator (matrix) is used to describe an ensemble of quantum states; in other words a classical *statistical mixture* of quantum states (kets) {|*φ_n_*〉} with probability distribution {*P_n_*} (∑*_n_P_n_*=1) as follows $\rho =\sum_{n}P_{n}|\phi_{n}\rangle \langle \phi_{n}|$, where |*φ_n_*〉 is a column-vector (ket), while 〈*φ_n_*| is a row-vector representing a Hermitian conjugate of ket, often called “bra”. In quantum information theory, the density matrix *ρ* can be used to determine the amount of information conveyed by the quantum state, *i.e.*, to compute the von Neumann entropy defined as $S\left(\rho \right)=Tr\left(\rho \log\rho \right)=-\sum_{i}\lambda_{i}\log_{2}\lambda ,$ where λ_i_ are the eigenvalues of the density matrix. The corresponding Shannon entropy can be calculated by $H=-\sum_{i}p_{i}\log_{2}p_{i},$ where *p_i_* is the probability of selecting the *i*-th vector from an ensemble of orthogonal vectors. Thus, the Shannon (classical) entropy is just a special case of the von Neumann (quantum) entropy, when the density matrix is diagonal. Let the composite system *C* be composed of quantum system *Q* and environment *E*. If the initial system-environment state was pure $|\psi_{QE}\rangle$ the corresponding density operator is defined as $\rho_{QS}=|\psi_{QE}\rangle \langle \psi_{QE}|$. By tracing-out the environment, we can determine the density operator of the system as $\rho =Tr_{E}|\psi_{QE}\rangle \langle \psi_{QE}|$. The presence of off-diagonal elements is commonly referred to as coherence. In a typical system in quantum physics, the off-diagonal elements will be averaged out and become eventually zeros, and we refer to this effect as decoherence. However, the biological systems are different (“wet, warm, and noisy”), and very exotic states get suppressed with biological system-environment interaction so that the biological system moves into the *preferred state*. This transformation (mapping) of the initial density operator of the system *ρ* to the final density operator of the biological system *ρ_f_*, represented by preferred states, can be described as $\xi :\rho \to \rho_{f}$, which is commonly referred to as the *superoperator* or *quantum operation*. The final density operator can be expressed in so called *operator-sum representation* as follows $\rho_{f}=\sum_{k}E_{k}\rho E_{k}^{†},$ where *E_k_* are known as the Kraus operators for the superoperator satisfying the normalization condition $\sum_{k}E_{k}E_{k}^{†}=I$ (where *I* is the identity operator). The Kraus operators for a memoryless (single-stage) quantum biological channel model, with codon nucleobase error probability as the parameter, are determined in our previous publication [8]. Here we determine the corresponding multi-stage quantum biological channel model.

**Figure 1:**
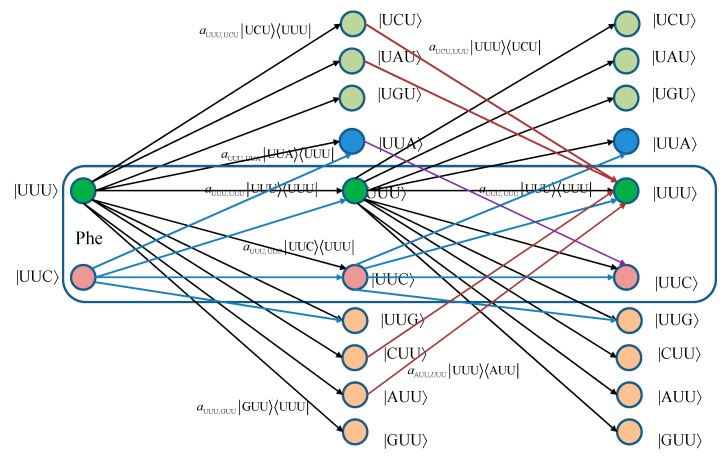
Two stages of a quantum biological channel model with memory for basekets corresponding to Phe. Only selected transitions have been shown to illustrate the model. The *a_m_*,*_n_* denotes a transition probability amplitude from baseket |*m*〉 to baseket |*n*〉, where *m* ∈ {UUU, UUC } and *n* could be any of 64 basekets. The probability amplitude is related to probability by *p_m_*,*_n_* = |*a_m_*,*_n_*|^2^. The Kraus operator *E_m_*,*_n_* is obtained as $E_{m,n}=a_{m,n}|n\rangle \langle m|.$

For convenience, we provide in Figure 1 two stages of the quantum biological channel model with memory for basekets corresponding to Phe. The error introduced by Kraus operator $a_{UUU,AUU}|AUU\rangle \langle UUU|$ leads to the change baseket representing Phe into the baseket representing Ile, where *a*_UUU,AUU_ denotes the probability amplitude for basket |UUU〉 to basket |AUU〉 transition. The corresponding probability is related to the probability amplitude by *p*_UUU,AUU_ =|*a*_UUU,AUU_|^2^. Now, by concatenating two stages of the transition diagram, the Krauss operator $a_{AUU,UUU}|UUU\rangle \langle AUU|$ in the second stage corrects the error introduced by the Kraus operator $a_{UUU,AUU}|AUU\rangle \langle UUU|$ in the first stage. What we have essentially just described is a Markov chain-like biological quantum channel model. Before we proceed further, we provide the brief review of the theory of Markovian chains [16].

The finite Markovian chain is a commonly used model in communication systems to describe both the sources and channels with memory. The Markovian stochastic process with a finite number of states {*S*} = {*S*_1_, …, *S_n_*} is characterized by transition probabilities *p_ij_* of moving from state *S_i_* to state *S_j_* (*i*, *j*=1, …, *n*). The Markov chain is the sequence of states with transitions governed by the following transition matrix:

(1) $$P=\left[p_{ij}\right]=\left[\begin{array}{cccc} p_{11} & p_{12} & \cdots & p_{1n}\\ p_{21} & p_{22} & \cdots & p_{2n}\\ \cdots & \cdots & \ddots & \vdots \\ p_{n1} & p_{n2} & \cdots & p_{nn} \end{array}\right],$$

where ∑*_j_* *p_ij_* = 1. The probability of reaching all states from initial states after *k*-steps can be determined by

(2) $$P^{\left(k\right)}=P^{\left(0\right)}P^{k},$$

where ***P***^(0)^ is a row-vector containing the probabilities of initial states.

For the regular Markov chain (the *k*-th stage transition matrix has only nonzero entries), the transition matrix converges to stationary transition matrix ***T*** with all rows identical to each other:

(3) $$T=\underset{k\to \infty }{\lim}P^{k}=\left[\begin{array}{cccc} t_{1} & t_{2} & \cdots & t_{n}\\ t_{1} & t_{2} & \cdots & t_{n}\\ \cdots & \cdots & \ddots & \vdots \\ t_{1} & t_{2} & \cdots & t_{n} \end{array}\right].$$

In addition, the following is valid:

(4) $$\underset{k\to \infty }{\lim}P^{\left(k\right)}=\underset{k\to \infty }{\lim}P^{\left(0\right)}P^{k}=P^{\left(0\right)}T=\left[\begin{array}{cccc} t_{1} & t_{2} & \cdots & t_{n} \end{array}\right],$$

so that we can find stationary probabilities of states (or equivalently solve for elements of ***T***) from equations

(5) $$\begin{array}{l} t_{1}=p_{11}t_{1}+p_{21}t_{2}+\cdots +p_{n1}t_{n}\\ t_{2}=p_{12}t_{1}+p_{22}t_{2}+\cdots +p_{n2}t_{n}\\ \vdots \\ t_{n}=p_{1n}t_{1}+p_{2n}t_{2}+\cdots +p_{nn}t_{n}\\ \sum_{i=1}^{n}t_{i}=1. \end{array}$$

The theory of Markov chains has already been used in biology to describe population processes. In particular the Leslie matrix has been used to describe the age-structured model of population growth in population ecology [21]. Markov chains have also been used in population genetics to describe the change in gene frequencies in small populations affected by genetic drift [22]. The Markov chain theory has also been used to study the complexity of the protein families [23].

**Figure 2:**
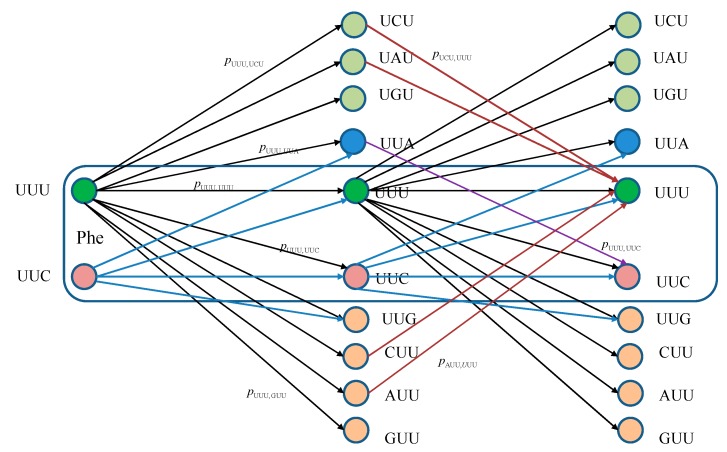
Two stages of a classical biological channel model with memory for basekets corresponding to Phe. Only selected transitions have been shown to illustrate the model. The *p_m_*_,*n*_ denotes the transition probability from state *m* ∈ {UUU, UUC} to state *n* (any of 64 basekets).

The equivalent classical biological channel model corresponding to Figure 1 is provided in Figure 2. As an illustration, the probability of moving from state UUU to UUC in two steps can be calculated as:

(6) $$p_{UUU,UUC}^{\left(2\right)}=p_{UUU,UUU}p_{UUU,UUC}+p_{UUU,UUC}p_{UUC,UUC}+p_{UUU,UUG}p_{UUG,UUC}+p_{UUU,UUA}p_{UUA,UUC},$$

which is consistent with Markovian chains theory described above.

**Figure 3:**
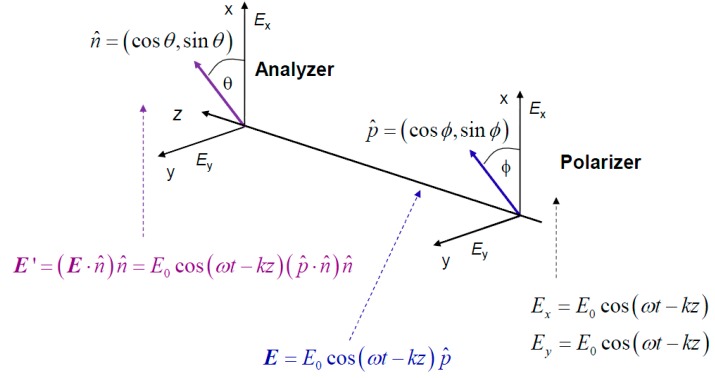
The study of the photon polarization by polarizer-analyzer ensemble.

Unfortunately, Markov chain theory is not consistent with quantum mechanics, as in sequential processes in quantum mechanics we need to multiply probability amplitudes instead [24]. To clarify this claim, we provide in Figure 3 the polarizer-analyzer ensemble. When an electromagnetic (EM) wave passes through the polarizer, it can be represented as a vector in the xOy plane transversal to the propagation direction *z*. The electric filed vector of EM wave can be written as:

(7) $$E=E_{0}\hat{p}\cos\left(\omega t-kz\right),$$

where $\hat{p}=\left(\cos\phi ,\sin\phi \right)$ is the polarization orientation unit vector with *φ* being an angle between the electrical field EM vector and x-axis. (*k* denotes the wave number.) If *φ*= 0 rad, the light is polarized along x-axis, while for *φ* = π/2 rad it is polarized along y-axis. After the analyzer, whose axis makes an angle *θ* with respect to x-axis, which can be represented by unit vector $\hat{n}=\left(\cos\theta ,\sin\theta \right)$, the output electric field is given by:

(8) $$\begin{array}{l} E\prime =\left(E\cdot \hat{n}\right)\hat{n}=E_{0}\cos\left(\omega t-kz\right)\left(\hat{p}\cdot \hat{n}\right)\hat{n}=E_{0}\cos\left(\omega t-kz\right)\left[\left(\cos\phi ,\sin\phi \right)\cdot \left(\cos\theta ,\sin\theta \right)\right]\hat{n}\\ \;=E_{0}\cos\left(\omega t-kz\right)\left[\cos\phi \cos\theta +\sin\phi \sin\theta \right]\hat{n}=E_{0}\cos\left(\omega t-kz\right)\cos\left(\phi -\theta \right)\hat{n}. \end{array}$$

The intensity of the electrical filed of EM wave at the output of analyzer can be written as:

(9) $$I\prime ={\left|E\prime \right|}^{2}=I\cos^{2}\left(\phi -\theta \right),$$

which is commonly referred to as Malus’ law. Classical physics prediction of total probability of a photon passing the polarizer-analyzer ensemble is given by:

(10) $$p_{tot}=\cos^{2}\phi \cos^{2}\theta +\sin^{2}\phi \sin^{2}\theta \neq \cos^{2}\left(\phi -\theta \right),$$

which is inconsistent with Malus’ law, given by Equation (9). In order to reconstruct the results from wave optics, the concept of probability amplitude that an angle α is detected as β, denoted as a(α→β), is introduced in quantum mechanics[24]. The probability is obtained as the squared magnitude of probability amplitude $p\left(\alpha \to \beta \right)={\left|a\left(\alpha \to \beta \right)\right|}^{2}.$ The basic principles of quantum mechanics tell us that we need to sum up the probability amplitudes for indistinguishable paths:

(11) $$a_{tot}=\cos\phi \cos\theta +\sin\phi \sin\theta =\cos\left(\phi -\theta \right).$$

The corresponding total probability is given by

(12) $$p_{tot}={\left|a_{tot}\right|}^{2}=\cos^{2}\left(\phi -\theta \right),$$

and this result is consistent with the Malus’ law.

We now apply this probability amplitude strategy to a quantum biological channel model with memory illustrated in Figure 1. As an illustration, let us determine the Kraus operator for moving from state |UUU〉 to state |UUC〉 in two steps, which will be denoted as $E_{UUU,UUC}^{\left(2\right)}$. Let us observe only a single nucleobase error per codon events. From quantum information processing theory [24] we know that, for serial (cascade) connection of gates, we need to multiply the corresponding operators, while for parallel connection of gates we need to sum-up the corresponding operators. Therefore, the corresponding Kraus operator can be obtained as:

(13) $$E_{UUU,UUC}^{\left(2\right)}=E_{UUU,UUC}E_{UUU,UUU}+E_{UUC,UUC}E_{UUU,UUC}+E_{UUC,UUG}E_{UUG,UUU}+E_{UUC,UUA}E_{UUA,UUU}.$$

Now by expressing the Kraus operators in terms of basekets we obtain:

(14) $$\begin{array}{l} E_{UUU,UUC}^{\left(2\right)}=a_{UUU,UUC}a_{UUU,UUU}\left(|UUU\rangle \langle UUU|\right)\left(|UUU\rangle \langle UUU|\right)+a_{UUC,UUC}a_{UUU,UUC}\left(|UUC\rangle \langle UUC|\right)\left(|UUC\rangle \langle UUU|\right)\\ \;+a_{UUC,UUG}a_{UUG,UUU}\left(|UUC\rangle \langle UUG|\right)\left(|UUG\rangle \langle UUU|\right)+a_{UUC,UUA}a_{UUA,UUU}\left(|UUC\rangle \langle UUA|\right)\left(|UUA\rangle \langle UUU|\right). \end{array}$$

By applying the associativity axiom we can re-write the previous equation as:

(15) $$\begin{array}{l} E_{UUU,UUC}^{\left(2\right)}=a_{UUU,UUC}a_{UUU,UUU}|UUU\rangle \langle UUU|UUU\rangle \langle UUU|+a_{UUC,UUC}a_{UUU,UUC}|UUC\rangle \langle UUC|UUC\rangle \langle UUU|\\ \;+a_{UUC,UUG}a_{UUG,UUU}|UUC\rangle \langle UUG|UUG\rangle \langle UUU|+a_{UUC,UUA}a_{UUA,UUU}|UUC\rangle \langle UUA|UUA\rangle \langle UUU|. \end{array}$$

From orthogonality principle we know that 〈UUU|UUU〉=〈UUC|UUC〉=〈UUG|UUG〉=〈UUA|UUA〉=1, so that we can re-write (15) as:

(16) $$\begin{array}{l} E_{UUU,UUC}^{\left(2\right)}=a_{UUU,UUC}a_{UUU,UUU}|UUU\rangle \langle UUU|+a_{UUC,UUC}a_{UUU,UUC}|UUC\rangle \langle UUU|\\ \;+a_{UUC,UUG}a_{UUG,UUU}|UUC\rangle \langle UUU|+a_{UUC,UUA}a_{UUA,UUU}|UUC\rangle \langle UUU|. \end{array}$$

From (16) we conclude that the probability amplitude $a_{UUU,UUC}^{\left(2\right)}$ can be calculated from the probability amplitudes of individual stages as follows:

(17) $$a_{UUU,UUC}^{\left(2\right)}=a_{UUU,UUC}a_{UUU,UUU}+a_{UUC,UUC}a_{UUU,UUC}+a_{UUC,UUG}a_{UUG,UUU}+a_{UUC,UUA}a_{UUA,UUU}.$$

The corresponding probability for moving from state |UUU〉 to state |UUC〉 in two steps will be:

(18) $$p_{UUU,UUC}^{\left(2\right)}={\left|a_{UUU,UUC}^{\left(2\right)}\right|}^{2}=|a_{UUU,UUC}a_{UUU,UUU}+a_{UUC,UUC}a_{UUU,UUC}+{a_{UUC,UUG}a_{UUG,UUU}+a_{UUC,UUA}a_{UUA,UUU}|}^{2},$$

which is clearly different from that obtained by classical Markov chain theory given by Equation (6).

As another illustration, the probability amplitude for moving from state |UUU〉 to state |UUU〉 in two steps can be determined as:

(19) $$\begin{array}{l} a_{UUU,UUU}^{\left(2\right)}=a_{UUU,UUU}a_{UUU,UUU}+a_{UUU,UUG}a_{UUG,UUU}+a_{UUU,UUC}a_{UUC,UUU}+a_{UUU,UUA}a_{UUA,UUU}\\ \;+a_{UUU,UGU}a_{UGU,UUU}+a_{UUU,UCU}a_{UCU,UUU}+a_{UUU,UAU}a_{UAU,UUU}\\ \;+a_{UUU,GUU}a_{GUU,UUU}+a_{UUU,CUU}a_{CUU,UUU}+a_{UUU,AUU}a_{AUU,UUU}. \end{array}$$

From a single-stage model we would expect to have only one probability amplitude. However, since a single nucleobase error in the first stage can be corrected for by the same nucleobase error in the second stage, the number of transitions in second stage gets increased to even nine. Based on discussion above, the probability amplitude transition matrix for the first stage can written as:

(20) $$A^{\left(1\right)}=A=\left[\begin{array}{cccc} a_{11} & a_{12} & \cdots & a_{1N}\\ a_{21} & a_{22} & \cdots & a_{2N}\\ \cdots & \cdots & \ddots & \vdots \\ a_{N1} & a_{N2} & \cdots & a_{NN} \end{array}\right],$$

where *a_m_*,*_n_* denotes the transition probability amplitude from baseket |*m*〉 to baseket |*n*〉, where *m*, *n* could be any of 64 basekets (the size of matrix is *N* × *N*, where *N* = 64). The corresponding probability amplitude is related to the probability by *p_m_*,*_n_* = |*a_m_*,*_n_*|^2^. The Kraus operator *E_m_*,*_n_* is obtained as $E_{m,n}=a_{m,n}|n\rangle \langle m|.$ Since the elements of the probability amplitude transition matrix are complex numbers, the matrix ***A*** is not stochastic. The *k*-th stage (step) transition probability matrix can be determined as:

(21) $$A^{\left(k\right)}≐\left[\begin{array}{cccc} a_{11}^{\left(k\right)} & a_{12}^{\left(k\right)} & \cdots & a_{1,N}^{\left(k\right)}\\ a_{21}^{\left(k\right)} & a_{22}^{\left(k\right)} & \cdots & a_{2,N}^{\left(k\right)}\\ \cdots & \cdots & \ddots & \vdots \\ a_{N,1}^{\left(k\right)} & a_{N,2}^{\left(k\right)} & \cdots & a_{N,N}^{\left(k\right)} \end{array}\right]=A^{\left(k-1\right)}A=\left[\begin{array}{cccc} a_{11}^{\left(k-1\right)} & a_{12}^{\left(k-1\right)} & \cdots & a_{1,N}^{\left(k-1\right)}\\ a_{21}^{\left(k-1\right)} & a_{22}^{\left(k-1\right)} & \cdots & a_{2,N}^{\left(k-1\right)}\\ \cdots & \cdots & \ddots & \vdots \\ a_{N,1}^{\left(k-1\right)} & a_{N,2}^{\left(k-1\right)} & \cdots & a_{N,N}^{\left(k-1\right)} \end{array}\right]\left[\begin{array}{cccc} a_{11} & a_{12} & \cdots & a_{1,N}\\ a_{21} & a_{22} & \cdots & a_{2,N}\\ \cdots & \cdots & \ddots & \vdots \\ a_{N,1} & a_{N,2} & \cdots & a_{N,N} \end{array}\right].$$

Clearly, from the matrix multiplication rule, the $a_{ij}^{\left(k\right)}$-th element is determined by:

(22) $$a_{m,n}^{\left(k\right)}=a_{m,1}^{\left(k-1\right)}a_{1,n}+a_{m,2}^{\left(k-1\right)}a_{2,n}+\cdots +a_{m,N}^{\left(k-1\right)}a_{N,n}=\sum_{l=1}^{N}a_{m,l}^{\left(k-1\right)}a_{l,n}.$$

Since $A^{\left(k\right)}=A^{\left(k-r\right)}A^{\left(r\right)};\;r=1,..,k-1$ the *k*-step (*k*-stage) transition probability amplitudes satisfy the Chapman–Kolmogorov equation:

(23) $$a_{m,n}^{\left(k\right)}=a_{m,1}^{\left(k-r\right)}a_{1,n}^{\left(r\right)}+a_{m,2}^{\left(k-r\right)}a_{2,n}^{\left(r\right)}+\cdots +a_{m,N}^{\left(k-r\right)}a_{N,n}^{\left(r\right)}=\sum_{l=1}^{N}a_{m,l}^{\left(k-r\right)}a_{l,n}^{\left(r\right)}.$$

The superoperator expressed in terms of Kraus operators after *k*-stages, namely $E_{m,n}^{\left(k\right)}=a_{m,n}^{\left(k\right)}|n\rangle \langle m|$, can be written as:

(24) $$\xi^{\left(k\right)}(\rho_{s})=\sum_{m,n}E_{m,n}^{\left(k\right)}\rho_{s}E_{m,n}^{(k)†},$$

where *ρ_s_* is the biological system initial density matrix.

We will now demonstrate, that this representation is quite general and that famous quantum master equation (QME), commonly used in quantum physics and quantum biology, is just a first order approximation of the operatorsum representation. Under the first order assumption, we can write:

(25) $$\rho \left(t+\delta t\right)=\rho (t)+O(\delta t)=E_{0}\rho E_{0}^{†}+\sum_{k=1,2,...}E_{k}\rho E_{k}^{†},$$

where

(26) $$E_{0}=I+O(\delta t)=I+(K-jH)\delta t+o(\delta t)\text{ and }E_{k}=L_{k}\sqrt{\delta t}+O(\sqrt{\delta t});$$

with *H* and *K* being the Hermitian operators, while *L_k_* are Lindblad operators. From the normalization condition we have that:

(27) $$\sum_{k=0,1,...}E_{k}E_{k}^{†}=I=I+2K\delta t+\sum_{k=1,2,...}L_{k}^{†}L_{k}\delta t+o(\delta t),$$

which indicates that

(28) $$K=-0.5\sum_{k=1,2,...}L_{k}^{†}L_{k}.$$

As *δt*→0, the Equation (25), after the substitution of (28) into (26) and then (26) into (25), becomes

(29) $$\frac{\partial \rho }{\partial t}=\left[-jH,\rho \right]+\sum_{k=1,2,...}\left[L_{k}\rho L_{k}^{†}-0.5\left\{L_{k}^{†}L_{k},\rho \right\}\right],$$

where we use {*A*, *B*} = *AB* + *BA* to denote the anticommutator. Therefore, the QME (29) is just the approximation of the operator-sum representation (24), when *δt*→0. (For simplicity, we omitted the reduced Plank constant ℏ=h/2π from the discussion above.) In other words, for Markovian approximation, the quantum channel model description given by the operator-sum representation and QME description are equivalent to each other. The particular use of representation is dictated by the biological problem at hand. Notice that superposition principle has not been used at all in deriving the operator sum representation (24), indicating that it is also applicable to cases when the linearity assumption of quantum mechanics is not valid [19,20].

Now we apply the Holevo**-**Schumacher-Westmoreland (HSW) theorem [24] to calculate the quantum biological channel capacity as follows:

(30) $$C^{\left(k\right)}\left(\xi^{\left(k\right)}\right)=\underset{\left\{p_{x},\rho_{x}\right\}}{\max}\left[S\left[\xi^{\left(k\right)}\left(\sum_{x}p_{x}\rho_{x}\right)\right]-\sum_{x}p_{x}S\left(\xi^{\left(k\right)}\left({\text{ρ}}_{x}\right)\right)\right],$$

where the maximization is performed over *p_x_* and ***ρ****_x_*. In (30) we use the ensemble {*p_x_*, ***ρ****_x_*} to denote the ensemble of density matrices corresponding to different amino acids. With *S*(•) we denoted the von Neumann entropy S(ρ). The quantum biological channel capacity describes the maximum amount of genetic information transferred from DNA to protein, and it is expressed in bits/residue. It can also be used as a figure of merit to determine the fitness and healthiness of the cell, tissue, organ, or organism as a whole. For instance, if several cells in an organ have a low cell channel capacity, but average channel capacity of organ, normalized per cell, is high, and close to the maximum possible, the corresponding organ is still in excellent condition. However, as the number of abnormal cells increases, the corresponding average channel capacity of the organ decreases and once a critical threshold is achieved the organ functionality gets affected. In this scenario, a cancer cell generating nonsense proteins is considered to have zero channel capacity. Additionally, the cells in either a senescence state or an apoptosis state have zero capacity. In this context, measuring biological channel capacity would be a more reliable measure of “real” aging than any specific chemical change, such as glycation, blood pressure, skin elasticity, *etc.* Reliable markers of aging are highly important, in particular brain aging markers. The biological channel capacity can be used as such relevant marker. By measuring the average number of bits/residue would provide a more accurate measure of the biological age of a tissue or an organ than any single chemical measure. This can help in an early diagnosis of the detrimental effects of aging.

Orgel noticed in reference [25] that translation errors, given that the translation process has lower fidelity than the replication process, decreases further fidelity of the translation process affecting, therefore, the gene expression machinery and, as such, contribute to the reduction of cell vitality, which is related to the aging process. In the unified aging model discussed in the introduction, this will modify the transition probabilities in the damage Markov chain-like model. Therefore, the models introduced in this paper are consistent with Orgel’s observations. In our previous article [8], we classify various types of quantum errors into several broad categories: (i) storage errors that occur in DNA itself as it represents an imperfect storage of genetic information, (ii) replication errors introduced during the DNA replication process, (iii) transcription errors introduced during DNA to mRNA transcription, and (iv) translation errors introduced during the translation process. Orgel’s errors are clearly of type (iv). Our proposed models above, allow us to consider the various sources of genetic error jointly, as it was done in this paper, or to consider them separately. In the second case, each stage in the Markov chain-like model needs to be split into four sub-stages, each sub-stage corresponding to one type of dominant errors.

**Figure 4:**
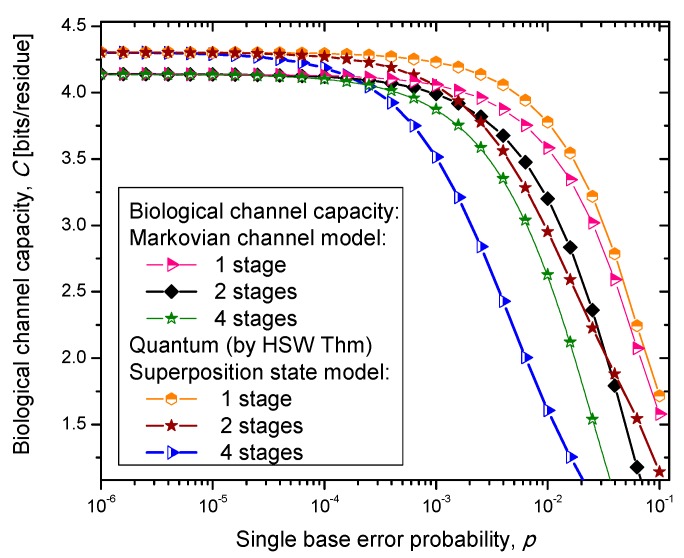
Channel capacity of quantum biological coherent state channel model with memory against the single base error probability.

**Figure 5:**
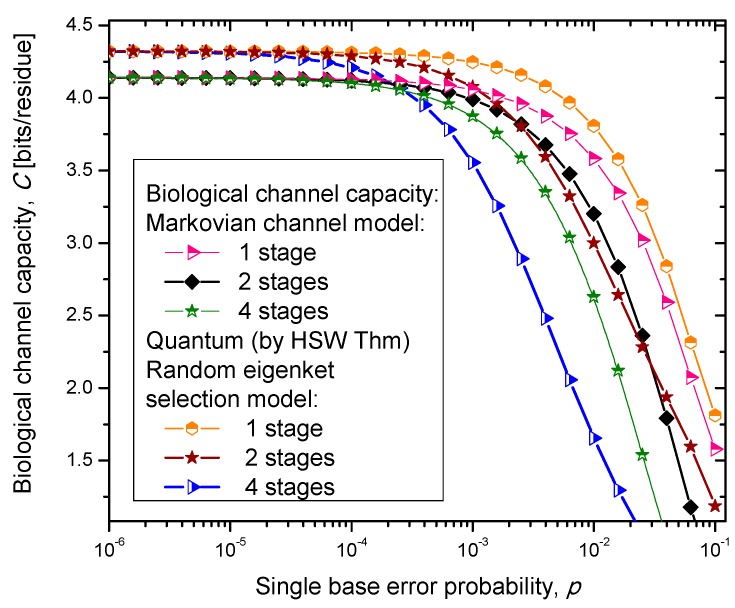
Channel capacity of quantum biological randomly selected eigenkets channel model with memory against the single base error probability.

**Figure 6:**
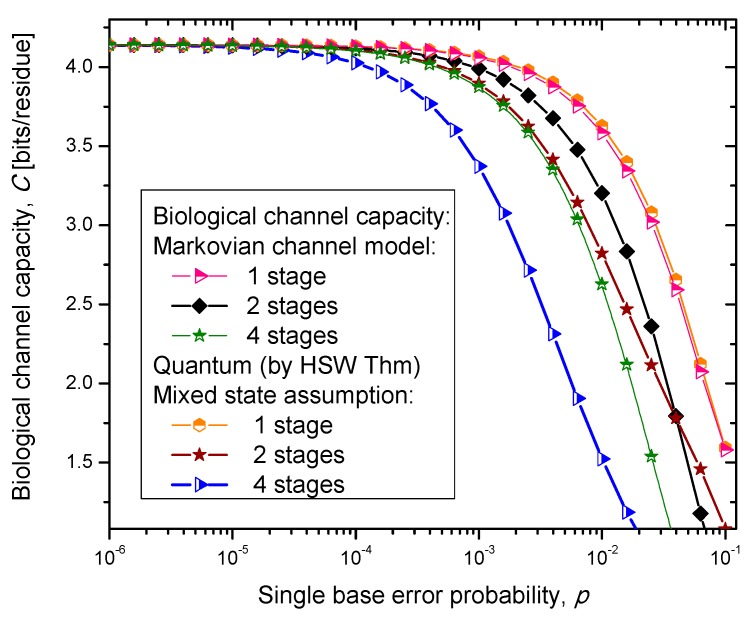
Channel capacity of quantum biological mixed state channel model with memory against the single base error probability.

Similarly, as in our previous paper [8], we consider three scenarios. In scenario (i), we assume that the codon state representing an amino acid state is a completely mixed state; a statistical mixture of basekets each occurring with the same probability. This scenario is essentially semi-classical. In scenario (ii), we assume that the amino acid codon-state is a superposition of eigenkets of corresponding Hamiltonian determined as described by Karafyllidis [14]. In case (iii), we select one of the amino acid codon eigenkets at random. Therefore, we perform the optimization only with respect to the prior probabilities of codons. The results of the calculation are summarized in Figure 4, Figure 5 and Figure 6, where we show biological channel capacities expressed in terms of bits/residue against the single nucleobase error probability. The general observations are: (i) the single stage quantum biological channel capacity for any of three models is always higher than corresponding classical biological channel capacity and (ii) the classical biological channel seems to more robust for more than one stages.

The quantum biological coherent state channel model has higher biological channel capacity than the corresponding classical Markovian model for two stages when the single base error probability is smaller than 1.8 × 10^−3^, than for four stages for *p* ≤ 2.3·10^−4^. On the other hand, the quantum biological random eigenkets selection model has higher biological channel capacity than the corresponding classical Markovian model for two stages when the single base error probability is smaller than 2.2 × 10^−3^, than for four stages for *p* ≤ 2.6 × 10^−4^. Since the mixed state channel model represents the classical statistical mixture of density operators, it is not surprising to see that it performs only slightly better than classical biological channel model. However, when the number of stages is larger than one it performs worse than the classical channel model except for very large single nucleobase error probabilities. When *p* ≥ 4 × 10^−2^, the two-stage mixed state model outperforms the classical model in terms of biological channel capacity.

From [26,27] we learned that the environment helps to suppress most of the exotic biological states and yields to the preferred state of the biological system. Therefore, the environmentally-induced decoherence is beneficial to the biological system. As such, it appears to make sense to study the hybrid quantum-classical biological channel models. Notice that these hybrid models are different from the synergetic model of DNA due to Koruga [28], which is essentially a tensor product of quantum and classical channel models. Our biological hybrid model is composed of *k_q_* quantum stages and *k_c_* classical stages. The density matrix after *k_q_* quantum stages can be described as:

(31) $$\rho^{\left(k_{q}\right)}=\sum_{m,n}E_{m,n}^{\left(k_{q}\right)}\rho_{s}E_{m,n}^{(k_{q})†},$$

where the Kraus operators are given by $E_{m,n}^{\left(k_{q}\right)}=a_{m,n}^{\left(k_{q}\right)}|n\rangle \langle m|,$ while the elements of probability amplitudes are determined based on Equation (21).

The Markovian transition probabilities matrix to be used in *k_c_* classical stages is determined by:

(32) $$P=\left[p_{ij}\right]=\left[\begin{array}{cccc} \frac{{\left|a_{11}^{\left(k_{q}\right)}\right|}^{2}}{\sum_{n=1}^{N}{\left|a_{1n}^{\left(k_{q}\right)}\right|}^{2}} & \frac{{\left|a_{12}^{\left(k_{q}\right)}\right|}^{2}}{\sum_{n=1}^{N}{\left|a_{1n}^{\left(k_{q}\right)}\right|}^{2}} & \cdots & \frac{{\left|a_{1,N}^{\left(k_{q}\right)}\right|}^{2}}{\sum_{n=1}^{N}{\left|a_{1n}^{\left(k_{q}\right)}\right|}^{2}}\\ \frac{{\left|a_{21}^{\left(k\right)}\right|}^{2}}{\sum_{n=1}^{N}{\left|a_{2n}^{\left(k_{q}\right)}\right|}^{2}} & \frac{{\left|a_{22}^{\left(k\right)}\right|}^{2}}{\sum_{n=1}^{N}{\left|a_{2n}^{\left(k_{q}\right)}\right|}^{2}} & \cdots & \frac{{\left|a_{2,N}^{\left(k\right)}\right|}^{2}}{\sum_{n=1}^{N}{\left|a_{2n}^{\left(k_{q}\right)}\right|}^{2}}\\ \cdots & \cdots & \ddots & \vdots \\ \frac{{\left|a_{N,1}^{\left(k\right)}\right|}^{2}}{\sum_{n=1}^{N}{\left|a_{Nn}^{\left(k_{q}\right)}\right|}^{2}} & \frac{\left|a_{N,2}^{\left(k\right)}\right|}{\sum_{n=1}^{N}{\left|a_{Nn}^{\left(k_{q}\right)}\right|}^{2}} & \cdots & \frac{{\left|a_{N,N}^{\left(k\right)}\right|}^{2}}{\sum_{n=1}^{N}{\left|a_{Nn}^{\left(k_{q}\right)}\right|}^{2}} \end{array}\right],$$

where the normalization per row ensures that the ***P***-matrix is stochastic. The Markovian transition probabilities matrix after *k_c_* classical steps is determined by:

(33) $$P^{\left(k_{c}\right)}=P^{k_{c}}.$$

The codon transition probabilities determined by (32) are employed to evaluate the classical biological channel capacity, defined as

**Figure 7:**
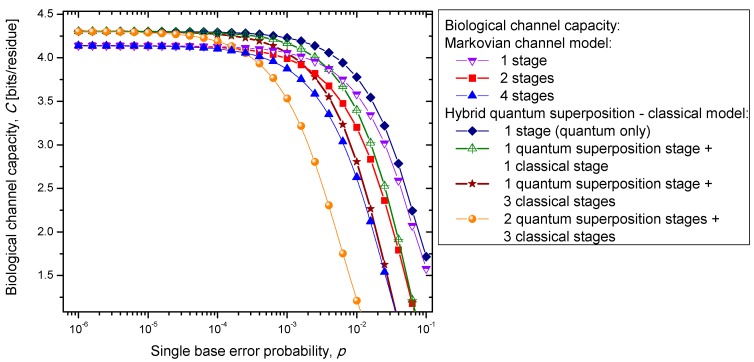
Channel capacity of hybrid biological coherent state—classical channel model with memory against the single base error probability.

(34) C=max[H(Y)−H(Y|X)],

where *H*(*Y*) and *H*(*Y*) stand for the biological Shannon (classical) channel input and output entropies, while *H*(*Y*|*X*) represents the conditional entropy of the biological channel output given the biological channel input *X*. The Shannon (classical) entropy of biological channel output and conditional entropy are defined respectively as:

(35) $$H(Y)=-\sum_{m,n}p(Y_{n}|X_{m})P(X_{m})\log_{2}[\sum_{m}p(Y_{n}|X_{m})p(X_{m})]$$

(36) $$H(Y|X)=-\sum_{m}\sum_{n}p(Y_{m}|X_{m})p(X_{m})\log_{2}P(Y_{n}|X_{m})$$

where we use {*p*(*X_i_*)} to denote the probability of occurrence of codons in DNA, and {*p*(*Y_j_*|*X_i_*)} to denote the conditional probabilities of the received codons {*Y_i_*} given the transmitted codons {*X*_i_}. The results of calculations are summarized in Figure 7. Clearly, the hybrid biological channel model with *k_q_* quantum stages and *k_c_* classical stages always has higher biological channel capacity than the Markovian classical channel model with *k* =*k_q_* + *k_c_* classical stages for all values of the single codon error probability. So it appears that the hybrid biological channel model is the most robust among different biological channel models described above. From these results we can conclude that it is beneficial for the biological system to be, initially, in a quantum state as it exhibits, then, a higher channel capacity, while the later transition into a classical state helps improve robustness against genetic noise.

The problem of aging is closely related to mutations. It is present in multicellular organisms. The single cell organisms do not really age in an ordinary sense. In single cell organisms, if the cell gets damaged, it either adapts or dies. The aging of multicellular organisms is due to change or loss of genetic information [5]. Namely, aging can be associated with the accumulation of mutation errors that eventually leads to partial loss of genetic information. In particular, the nuclear DNA damage can contribute either directly (by increased cell dysfunction) or indirectly (through apoptosis or cellular senescence) to the aging process. In tissues where the cells divide frequently, the somatic mutations are not that dangerous if the rate of mutations is sufficiently low. However, nerve or brain cells stop their replication at a certain age, and a great portion of somatic mutation on these cells is responsible for aging. Additionally, the somatic mutations in non-dividing cells are responsible for the aging process due to senescence/depletion. Different stages of Markovian-like biological channel models described above can be considered as different stages in life as well. As the time progresses, the number of possible error-event paths in genetic information increases, leading directly to the change of primary structure of the key enzymes. Since the key enzymes necessary to regulate metabolic processes in an organism are affected by the accumulation of random errors, the organism’s metabolism as a whole gets affected as well. The mutations could also be caused by mutagens and carcinogens. Well known examples of exogenous mutagens include intercalators (EtBr whose molecule can get inserted between the planar nucleobases of DNA and deform the structure of DNA) and base analogs (5-bromouracil (5-BU) which pretend to be a nucleobase but act differently. Namely, the 5-BU can replace uracil, but can have both keto and enol forms, which act differently. The keto form of BU pairs very well with A, while enol form pairs very well with G, instead, introducing additional mutations. Therefore, induced mutations increase the codon error probability. From Figure 7 it is evident that the increase of the single base error probability above 10^−4^ leads to a dramatic decrease in genetic information for multistage models. In conclusion, the combination of multistage error events and codon error probability increases can be considered as the main sources of the aging process.

This theory of aging is known as damage/error theory of aging. Another relevant theory of aging is programmed theory of aging [18], in which the aging process is dictated by a biological clock. The control regulation, in this model, is dependent on gene regulation affecting the system responsible for maintenance, repair mechanisms, and defense responses. These two theories are not mutually exclusive, but might interact in a complex way as indicated by Jin [18]. Additional details on this topic are provided in concluding remarks section, Section 4.

## 3. Quantum Biological Channel Capacity Evolution through Generations

**Figure 8:**
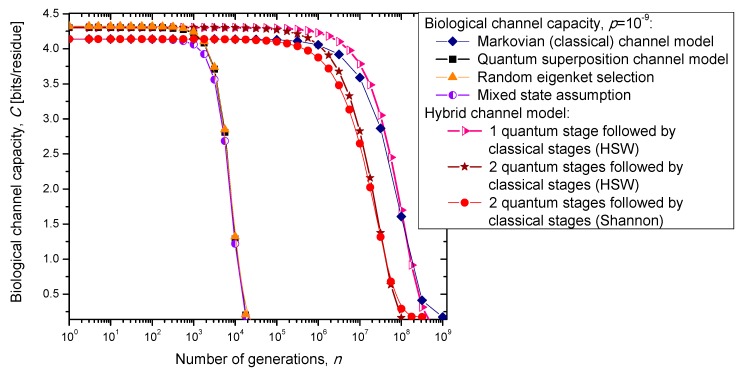
Evolution of biological channel capacity through generations when the base error probability is set to 10^−9^.

**Figure 9:**
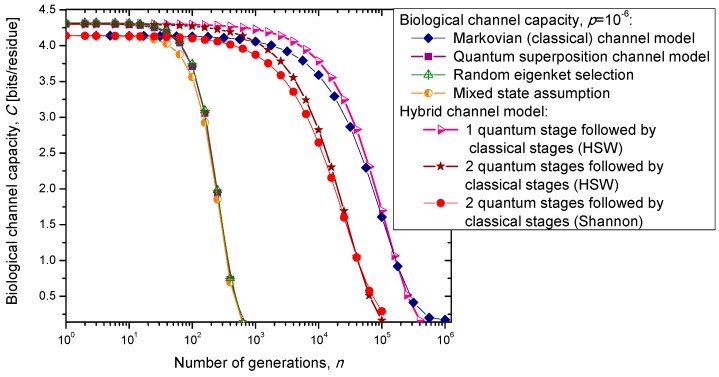
Evolution of biological channel capacity through generations when the base error probability is set to 10^−6^.

**Figure 10:**
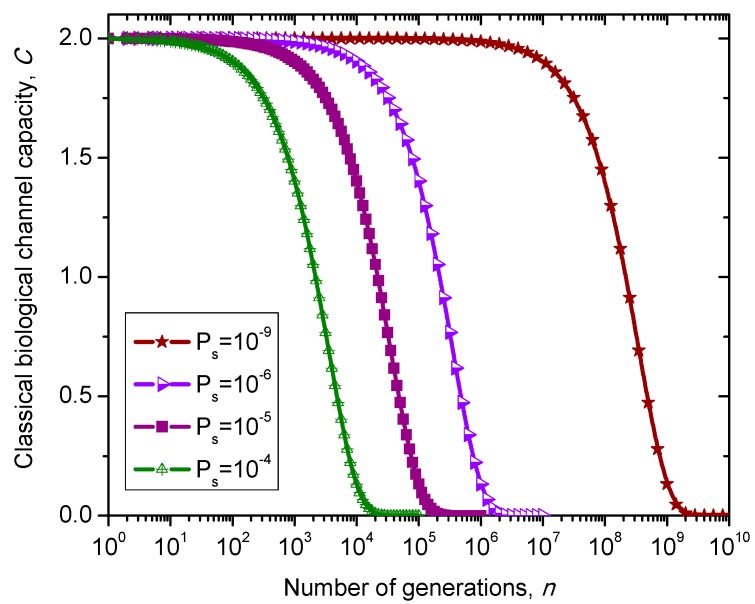
Evolution of non-protein coding DNA biological classical channel capacity through generations based on 4-ary SC-inspired Markovian process for different nucleotide error probabilities *P_s_*.

In the previous section, we have described the classical Markovian model and the quantum Markov-like model to describe mutations and aging. The same model is also applicable in describing genome preservation through generations. The results of the calculation of evolution of biological channel capacity through generations, for various quantum and classical models described in Section 2, are summarized in Figure 8 and Figure 9 for different base error probabilities *p*: 10^−9^ in Figure 8 and 10^−6^ in Figure 9. The classical Markovian model shows much better robustness through generations compared to various quantum Markovian-like models, except the mixed state model. On the other hand, quantum superposition and random eigenket selection Markovian-like models exhibit higher biological channel capacity for up to 1455 generations when the base error probability is 10^−9^ (see Figure 8). However, for typical base error probability (around 10^−6^), the random eigenket selection model exhibits higher biological channel capacity for up to 49 generations only (see Figure 9).

The hybrid quantum-classical model, also described in Section 2, exhibits better robustness (through generations) compared to purely quantum Markovian-like models. If the hybrid channel model preserves the coherence over generations, we have to use the HSW theorem to calculate the quantum biological channel capacity, based on Equation (30). In this case, as shown in Figure 8 and Figure 9, not only that the system is more robust than the corresponding classical model, but also exhibits higher biological channel capacity. If, however, the coherence is not preserved, but the system faces quantum to classical transition, in similar fashion as described by Zurek [29], we have to use the classical information theory concepts, in particular Equations (34)–(36) from the previous section in calculating the biological channel capacity. Such a system exhibits lower than classical model biological channel capacity, but has similar robustness of genome information through generations when compared to the classical Markovian channel model.

Some other models to describe the evolution of genetic information through generations include the *M*-ary symmetric channel model, where *M* = 4 (see reference [16]), and the Kimura model [17]. Let *X* = {*x*_0_, …, *x_I_*_-1_} and *Y* = {*y*_0_, …, *y_J_*_-1_} denote the input and output alphabets, respectively. Additionally, let *p*(*y_j_*|*x_i_*) denote the transition probability Pr(*Y* = *y_j_*|*X*=*x_i_*) and *P_s_* denote symbol error probability. Then, for the *M*-ary symmetric channel (MSC), the transition probability is given by *p*(*y_j_*|*x_i_*) = *P_s_*/(*M*−1) when *i* ≠ *j* and *p*(*y_i_*|*x_i_*) = 1−*P_s_*. The 4-ary symmetric channel model can also be used to develop Markovian model, whose transition probability matrix is given by:

(37) $$\begin{array}{l} \;\begin{array}{cccc} T & \;C & \;A & \;G \end{array}\\ P_{4SC}=\begin{array}{c} T\\ C\\ A\\ G \end{array}\left[\begin{array}{cccc} 1-P_{s} & P_{s}/3 & P_{s}/3 & P_{s}/3\\ P_{s}/3 & 1-P_{s} & P_{s}/3 & P_{s}/3\\ P_{s}/3 & P_{s}/3 & 1-P_{s} & P_{s}/3\\ P_{s}/3 & P_{s}/3 & P_{s}/3 & 1-P_{s} \end{array}\right]. \end{array}$$

The transition matrix of 4-ary symmetric channel (SC)-based Markovian process, corresponding to the *n*-th generation, is determined by $P_{4SC}^{n}$. The classical biological channel capacity is calculated then by Equations (34)–(36) from the previous section. The results of calculations are summarized in Figure 10 for different nucleotide (symbol) error probabilities *P_s_*. Since this model does not distinguish between exons and introns than corresponding channel capacity, expressed in bits/symbol, can be called non-protein coding DNA biological channel capacity and, strictly speaking, is not comparable to classical and quantum models described above, with results summarized in Figure 8 and Figure 9. Moreover, *P_s_* is the nucleotide error probability while *p* in Figure 8 and Figure 9 is the single base error probability per codon.

The Kimura model [17] can be considered as a generalization of 4-ary symmetric channel model whose transition matrix is given as follows:

(38) $$\begin{array}{l} \;\begin{array}{cccc} \;T & \;C & \;A & \;G \end{array}\\ P_{\text{Kimura}}=\begin{array}{c} T\\ C\\ A\\ G \end{array}\left[\begin{array}{cccc} 1-P_{s} & \gamma P_{s}/3 & P_{s}\gamma /3 & (1-2\gamma /3)P_{s}\\ \gamma P_{s}/3 & 1-P_{s} & (1-2\gamma /3)P_{s} & \gamma P_{s}/3\\ \gamma P_{s}/3 & (1-2\gamma /3)P_{s} & 1-P_{s} & \gamma P_{s}/3\\ (1-2\gamma /3)P_{s} & \gamma P_{s}/3 & \gamma P_{s}/3 & 1-P_{s} \end{array}\right], \end{array}$$

where γ∈[0,3/2] is the parameter of the Kimura model with typical values ranging between 0.07 and 0.79. Clearly, for γ = 1 the Kimura model reduces to 4-ary symmetric channel.

What is interesting about the Kimura model is it can distinguish between transitions and transversions during the base substitution mutations. In transitions, the category of bases during base substitution is preserved and the transition probability in this case is (1-2*γ*/3)*P_s_*. On the other hand, in transversions, the base category gets changed, from purine to pyrimidine type and vice versa, and the transition probability in this case is *γP_s_*/3. The transition matrix of the Kimura model-based Markovian process, corresponding to the *n*-th generation, is determined by $P_{\text{Kimura}}^{n}$.

**Figure 11:**
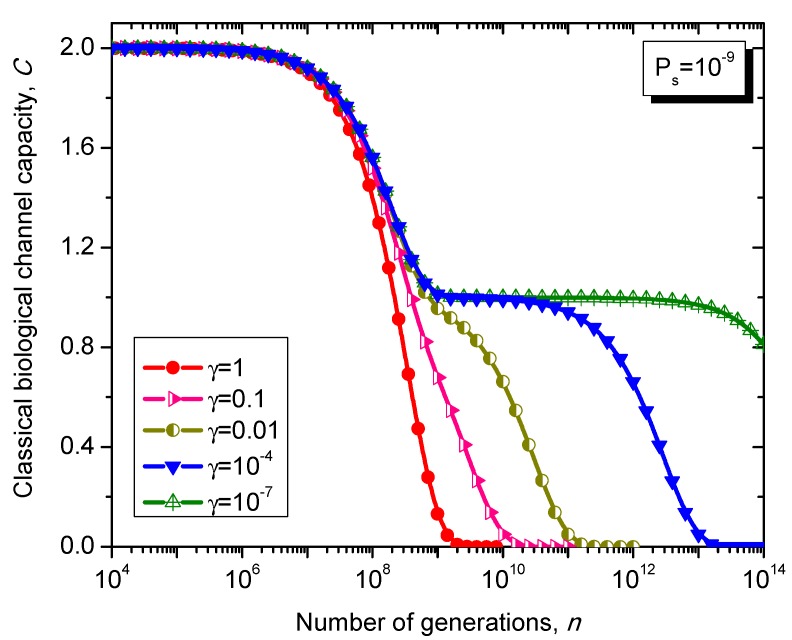
Evolution of non-protein coding DNA classical biological channel capacity through generations based on the Kimura model-inspired Markovian process for different values of parameter γ and symbol error probability set to 10^−9^.

**Figure 12:**
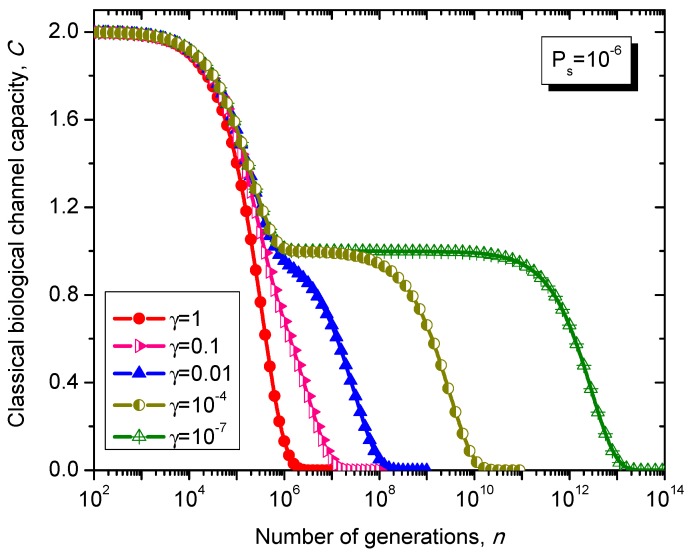
Evolution of non-protein coding DNA classical biological channel capacity through generations based on the Kimura model-inspired Markovian process for different values of parameter γ and symbol error probability set to 10^−6^.

The classical biological channel capacity, expressed in bits/symbol, is calculated then by Equations (34)–(36) from the previous section. The results of calculations are summarized in Figure 11 and Figure 12, for different nucleotide (symbol) error probabilities *P_s_*. In each figure the Kimura parameter γ is used as the parameter. Again, since *P_s_* is here nucleotide error probability while *p* in Figure 8 and Figure 9 is the single base error probability per codon the results shown in Figure 11 and Figure 12 are not really comparable against those shown in Figure 8 and Figure 9.

## 4. Concluding Remarks

This paper represents the continuation of our previous paper [8], in which the operator-sum representation of biological channel based on codon basekets and the corresponding quantum channel model have been derived. This memoryless quantum channel model has been extended in this paper to be able to describe the propagation of mutation errors in time, the process of aging, and evolution of genetic information through generations. The extended channel model is capable of accurately describing the process of the creation of spontaneous, induced, and adaptive mutations and their propagation in time, as well as the process of aging. The following three illustrative models, applicable in deferent regimes, have been proposed: (i) Markovian classical model, (ii) Markovian-like quantum model, and (iii) hybrid quantum-classical model. The proposed models have been applied to study aging and the evolution of quantum biological channel capacity through generations. The key differences of these models against the conventional multilevel symmetric channel-based Markovian model and the Kimura model-based Markovian process have been discussed. As we have shown in Section 2, these models are quite general and applicable to many open problems in biology, not only to biological channel capacity, representing the main focus of the paper. We have demonstrated that the famous quantum master equation approach, commonly used to describe different biological process, is just the first-order approximation of the proposed quantum Markov chain-like model, when the observation interval tends to zero. This indicates that the proposed models are more accurate and more general.

The proposed Markovian-like quantum biological model is applicable not only to damage/error models of aging, but also to time-dependent programed theories of aging; in particular the programmed senescence theory [18,30]. The proposed Markovian-like quantum channel model can also be used to unify damage and programmed senescence theories of aging as follows: namely, the life cycle can be represented in several stages, and the transition from stage to stage is determined by particular genes’ activation and deactivation. This transition from previous to next life stages is characterized by the increase in base error probability as well as changes in transition probability amplitudes *a_m_*_,*n*_ among codon-eigenkets. The senescence stage is defined as the stage when age-associated phenomena are visible, when the Markov-like quantum channel model is used. This unifying model is also applicable to describe the evolution of genetic information through generations.

In derivation of the operator-sum representation of the Markovian-like quantum biological channel model, the superposition principle has not been used at all, indicating that this model is also applicable to cases when the linearity assumption of quantum mechanics is not valid [19]. It is interesting to notice that, when the single codon error probability exceeds a certain threshold value, biological channel capacity decreases exponentially, which is consistent with paper by Melkikh [20].
